# Assessment of worm control practices recommended by equine veterinarians in Australia

**DOI:** 10.3389/fvets.2023.1305360

**Published:** 2023-11-03

**Authors:** Ghazanfar Abbas, Mark A. Stevenson, Jenni Bauquier, Anne Beasley, Caroline Jacobson, Charles El-Hage, Edwina J. A. Wilkes, Peter Carrigan, Lucy Cudmore, John Hurley, Ian Beveridge, Martin K. Nielsen, Kristopher J. Hughes, Abdul Jabbar

**Affiliations:** ^1^Melbourne Veterinary School, The University of Melbourne, Werribee, VIC, Australia; ^2^School of Agriculture and Food Sustainability, University of Queensland, Gatton, QLD, Australia; ^3^Centre for Animal Production and Health, Murdoch University, Murdoch, WA, Australia; ^4^Racing Victoria, Flemington, VIC, Australia; ^5^Scone Equine Hospital, Scone, NSW, Australia; ^6^Swettenham Stud, Nagambie, VIC, Australia; ^7^M.H. Gluck Equine Research Center, Department of Veterinary Science, University of Kentucky, Lexington, KY, United States; ^8^School of Agricultural, Environmental and Veterinary Sciences, Charles Sturt University, Wagga Wagga, NSW, Australia

**Keywords:** questionnaire survey, equine veterinarians, thoroughbred, worm control, Australia

## Abstract

This study aimed to assess Australian veterinarians’ knowledge, perceptions and treatment strategies for worm control in horses with an online questionnaire. The questionnaire comprised 64 questions covering various aspects of: (i) veterinary practice; (ii) the veterinarian’s knowledge of gastrointestinal nematodes (GINs) and the importance of parasites in different age groups of horses; (iii) the diagnosis and control of worms; (iv) anthelmintics and anthelmintic resistance (AR); (v) grazing management; and (vi) the means of communication and the discussion between veterinarians and their clients regarding worm control. Following a pilot survey, a link for the questionnaire survey was sent to all (*n* = 1,148) registered members of Equine Veterinarians Australia in April 2020. The response rate for the questionnaire was 10% (118 of 1,148). The findings of this study illustrate veterinarians’ good understanding of aspects of equine parasites, including control. However, respondents mainly recommended frequent, interval-based prophylactic deworming in young horses, and only 40% (96 of 239) diagnosed GIN infections based on faecal egg count (FEC) results in all age groups of horses. Furthermore, only 27% (88 of 330) of the respondents made deworming decisions based on FECs. Most of the respondents recommended macrocyclic lactones (MLs) for all age groups of horses (71%, 481 of 677), and the most frequently used method to calculate the dose of anthelmintics was by estimating the weight of animals visually (53%, 63 of 118). Although the majority of respondents (97%, 115 of 118) perceived AR to be a critical issue in managing worms in horses, 58% (67 of 118) of them were unaware of the status of AR on their clients’ properties. Forty-two percent (50 of 118) of the respondents perceived the presence of AR in worms, including pinworms (16%), strongylins (15%), species of *Draschia* and *Habronema* (6%), *Strongyloides westeri* (2%) and tapeworms (1%). Twenty-seven percent (32 of 118) of the respondents rarely discussed equine worm control practices with their clients. This study provides insights into the perception and worm control practices recommended by Australian veterinarians to manage equine parasites. The findings highlight the importance of continued education and awareness of AR, and the use of non-chemical methods as well as consideration of the legislation of prescription-only use of anthelmintics based on FECs to achieve sustainable control of GINs in Australian horses.

## 1. Introduction

Gastrointestinal nematodes (GINs) are one of the serious threats to horse health and welfare globally (1), with ascarids (*Parascaris* spp.) being important intestinal nematodes in foals, and cyathostomins ubiquitously infecting all age groups of horses (2). Broad-spectrum anthelmintics have been used for six decades to control GINs in managed horses (3, 4). However, the frequent, prophylactic and indiscriminate interval-based deworming of horses has resulted in the emergence of resistance in ascarid and strongylid nematodes against the commonly used anthelmintics such as benzimidazoles (BZs), tetrahydropyrimidines (THPs) and macrocyclic lactones (MLs) (5, 6). In Australia, anthelmintic resistance (AR) in the GINs of horses is not dissimilar to other parts of the world. For example, multiple studies assessing the efficacy of BZs, MLs, and THPs against GINs have reported AR in *Parascaris* spp. (7–10) and cyathostomins (10–12) of Australian Standardbred and Thoroughbred horses. As no new anthelmintic drug classes are likely to be developed for use in horses, adopting appropriate worm control practices to promote the judicious use of anthelmintics to achieve sustainable control of GINs is essential.

Numerous veterinary and veterinary parasitology organisations and equine parasite control advisory panels have established guidelines to prevent and reduce the development of AR in GINs of horses globally. The American Association of Equine Practitioners (AAEP) (13) and European Scientific Counsel Companion Animal Parasites (ESCCAP: https://www.esccap.org/life-cycles/gl8/) have recommended guidelines for parasite control in horses. However, expert recommendations are not always followed by the end-users (i.e., horse managers and veterinarians) and similar results have been found in sociological studies on understanding the low uptake of deworming strategies in small ruminants in France (14) and Morocco (15). Furthermore, several surveys aimed at assessing worm control practices used by horse managers in Australia (9, 10, 16), New Zealand (17, 18), Europe (19–23), the UK (24, 25), and the USA (26, 27) showed the widespread use of interval-based deworming and limited use of faecal egg counts (FECs) for deworming decisions and easy access to anthelmintic from farm supplies stores in some countries. This complex situation could be addressed by the active involvement of veterinarians in managing horse parasites, as these professionals should play a crucial role in promoting evidence-based worm control practices by educating horse owners and other industry stakeholders about the judicious use of anthelmintics and the impact of resistant GINs on the health and welfare of their animals. The need for veterinarians to be involved in horse parasite management was reported in studies of European horse owners (22, 24, 28) where frequent and systematic drenching schemes with limited use of FECs were determined.

In Denmark, implementation of a prescription-only framework, where anthelmintics are prescribed after FEC results, resulted in repositioning veterinarians as advisors while decreasing the number of treatments per horse each year (29). Recently, Becher et al. (30) conducted a multinational survey to compare equine parasite control strategies employed in some European countries (Austria, Denmark, Germany, and the Netherlands) where prescription-only restrictions of anthelmintic usage by law had been implemented, and the USA, where all anthelmintic products continue to be available over the counter. The authors found that Danish respondents used substantially more FECs and fewer anthelmintic treatments per horse per year than participants from the other four countries, indicating that more stringent guidelines do not necessarily have uniform outcomes across countries. For instance, a recent study illustrated that a targeted treatment approach in equids in tropical conditions was more costly than routine blanket deworming due to consistently high FECs (31). Therefore, the current knowledge of worm control practices in horses points to the need for improved and sustained education of both horse owners, and their veterinarians, to ensure the adoption of evidence-based control of equine parasites.

To date, very little is known about the level of knowledge of parasites and management recommendations made by practising veterinarians, as only a small number of studies addressing this issue have been conducted globally (29, 32, 33). In Australia, there are no national guidelines for the control of horse parasites and recent studies have shown that the majority of horse managers are using interval-based deworming as the primary worm control practice (10) and there is widespread resistance in ascarid and cyathostomins against commonly used anthelmintics (10, 12). As veterinarians are essential stakeholders, it is critical to ascertain their knowledge of parasites and the equine parasite management strategies they are recommending, to better understand the starting point for the development of tailored guidelines for the Australian equine industry. Therefore, this study aimed to understand Australian veterinarians’ knowledge, perceptions and treatment strategies for worm control in horses using a national online questionnaire survey to identify barriers to evidence-based parasite control.

## 2. Materials and methods

### 2.1. Study population

The target population for this study was veterinarians in Australia registered with Equine Veterinarians Australia (EVA) – a special interest group of the Australian Veterinary Association. The EVA was formed in 1971 and has more than 1,000 members in Australia, England, Hong Kong, New Zealand, Singapore, South Africa and the USA. A key role of the EVA is to provide advice and information to the people who care for horses to ensure that Australian horses enjoy the best possible health and welfare. The group maintains strong links with equine organisations and horse owners, supporting its members’ need to remain at the forefront of equine veterinary medicine. The group also represents members’ interests to governments and equine industry organisations.^1^

### 2.2. Questionnaire

A questionnaire survey was devloped and administered using an online programme, Research Electronic Data Capture^2^ following the approval of the Human Ethics Committee (Ethics ID 13193) of the University of Melbourne. Participation in the survey was voluntary. The questionnaire (see Supplementary material – Questionnaire) comprised 64 questions, including 57 closed-ended questions with multiple choices, Likert scale and yes/no options, and seven open-ended questions where respondents were asked to provide descriptive information or a number. A branching logic was used between various questions linked to each other.

The questionnaire comprised questions about: (i) veterinary practice description (e.g., location, number of veterinarians, gender, qualification(s), and experience); (ii) veterinarians’ knowledge of GINs and parasite importance in different age groups of horses; (iii) the diagnosis and control of worms; (iv) anthelmintics and AR; (v) grazing management; and (vi) means of communication and the discussion regarding worm control between veterinarians and their clients. The questions regarding deworming strategy, type of dewormers used, and the rotation of anthelmintics were stratified by horse age groups, including foals/weanlings (up to 1 year of age), juveniles (1–3 years) and adults (> 3 years, including mares, stallions, and geldings). Additionally, horses were further categorised into foals (up to 6 months), weanlings (6–12 months), juveniles (1–3 years) and mares, stallions and geldings (> 3 years) for questions about the number of horses on the property, groups of horses susceptible to the risk of worms, deworming frequency and grazing management both in and outside the breeding season.

Following the pilot survey involving 14 veterinarians, all registered EVA members (*n* = 1,148) were invited to participate in the study through an email (April 2020) with a link to the online questionnaire from the head office of the EVA. Additionally, the survey link was distributed by the state EVA branches and posted on social media platforms (i.e., Twitter and Facebook). The questionnaire remained open until mid-2021 and the EVA head office sent three reminders.

Respondents could submit the questionnaire anonymously but were asked to enter the four digits of their postal code to allow a general assessment of the geographical distribution of respondents.

### 2.3. Data analyses

Questionnaire data were exported to Microsoft Excel (2016) and checked for completeness and implausible values. Analyses were performed using R version 4.2.2 (34) and GraphPad Prism version 10.0.1 for Windows^3^ was used to create graphs. Data were included in the final dataset if they were either recorded as complete (*n* = 90) by the survey software or were partially completed with >40% of questions responded (*n* = 28). Data were checked for errors and free text comments and coded into further categories where appropriate. Some categories were combined and/or re-categorised where questions were provided as open-ended “other” options to reduce the number of variables for analyses.

The patterns of missingness in the data were imputed by the multiple imputation (MI) method using the contributed MICE package (35) in R. Missing values for all of the categorical and continuous questionnaire response variables were imputed using a classification and regression tree (CART) method. Following imputation, descriptive analyses were carried out on all questions. Fisher’s exact test was used to test for a statistically significant difference in the proportions of respondents recommending grazing management practices during the breeding and non-breeding seasons (36).

## 3. Results

### 3.1. Demography of respondents

One hundred eighty-nine respondents clicked the survey link, resulting in 90 complete and 99 incomplete responses. Based on the appraisal of incomplete questionnaires, 28 fulfilled the criteria for inclusion (i.e., completed >40% of the questionnaire and answered the main question on farm demographics, use of diagnostics and dewormers) and were included in data analyses after adding the missing values. Hence, the response rate for the questionnaire was 10% (118 of 1,148).

The response varied across different states of Australia, with the maximum responses from EVA members based in Victoria (39%) followed by New South Wales (24%), Queensland (18%), Western Australia (9%), South Australia (7%) and Tasmania (3%). The majority of respondents were females (61%, proportion 72 of 118), located in rural areas (60%, 74 of 124), equine-only practitioners (65%, 77 of 118), having their own practice (45%, 53 of 118) or employee at another practice (30%, 35 of 118) and had an experience of 11 to 20 years (28%, 33 of 118) (Table 1). Respondents from equine practices indicated that, on average, six (median 3) veterinarians were employed in the practice (Table 2). Most of the respondents had not additional qualifications to their Bachelor of Veterinary Science or Doctor of Veterinary Medicine degrees (56%, 66 of 118), whereas 23% (27 of 118) and 11% (13 of 118) had specialist qualifications, Masters, PhD and/or fellowships, respectively. The respondents were treating various types of horses, including pleasure (22%, 72 of 327), performance/sport (20%, 66 of 327), Thoroughbred racing (14%, 47 of 327), and Thoroughbred breeding (13%, 43 of 327) (Table 1). The maximum number of horses examined by the respondents in the last year were adults (*n* = 361 and 515) followed by juveniles (*n* = 186 and 203) and foals or weanlings (*n* = 106 and 95) in the breeding and non-breeding seasons, respectively (Table 2).

**Table 1:** Demographic information of respondents and the practices that participated in the survey.

Question (total responses)	Levels	Percentage (counts)
Gender (*n* = 118)	Female	61 (72)
	Male	39 (46)
Qualification (*n* = 118)	BVS/DVM	56 (66)
	Professional colleges fellowship	23 (27)
	Masters/PhD and fellowship	11 (13)
	Masters/PhD	8 (9)
	Other	3 (3)
Practice location (*n* = 124)^a^	Rural	60 (74)
	Peri-urban	28 (35)
	Urban	11 (14)
	Other (Australia wide)	1 (1)
Practice type (*n* = 118)	Equine only	65 (77)
	General/mixed practice	26 (31)
	Equine and production animals	3 (4)
	Other	5 (6)
Horses examined (*n* = 327)^a^	Pleasure	22 (72)
	Performance/sport	20 (66)
	Thoroughbred racing	14 (47)
	Thoroughbred breeding	13 (43)
	Stock horses	11 (35)
	Other breeding	10 (31)
	Standardbred racing	9 (29)
	Other	1 (4)
Role (*n* = 118)	Practice owner	45 (53)
	Practice employee (≥ 2 years)	30 (35)
	Equine specialist	13 (15)
	Practice employee (≤ 2 years – a recent graduate)	5 (6)
	Equine resident	3 (4)
	Other	4 (5)
Experience (*n* = 118)	<1 year	2 (2)
	1–2 years	7 (8)
	3–5 years	15 (16)
	6–10 years	16 (19)
	11–20 years	28 (33)
	21–30 years	16 (19)
	31–40 years	9 (11)
	>40 years	9 (10)

a Selection of multiple options was possible for these questions.

**Table 2:** Knowledge of respondents about horse parasites and the number of horses under their care in the last year (*n* = 118) that participated in the survey.

Question	Mean	Median (Q1, Q3)	Min, Max
Equine parasite knowledge	60	64 (50, 75)	14, 99
Full-time equivalent veterinarians	6	3 (1, 8)	0, 40
No. of horses you examined in the last 12 months	1,030	500 (150, 1,000)	2, 5,000
Breeding season			
Foals/Weanlings (up to 1 year)	106	40 (5, 100)	0, 1,500
Juveniles (1–3 years)	186	50 (14, 200)	0, 2,000
Adults (>3 years)	361	200 (75, 460)	0, 3,500
Non-breeding season			
Foals/Weanlings	95	38 (5, 100)	0, 1,500
Juveniles	203	50 (16, 200)	0, 2,000
Adults	515	200 (60, 713)	0, 3,500

Q1, first quartile; Q3, third quartile; Min, minimum; Max, maximum.

### 3.2. Knowledge about horse worms

**Figure 1:**
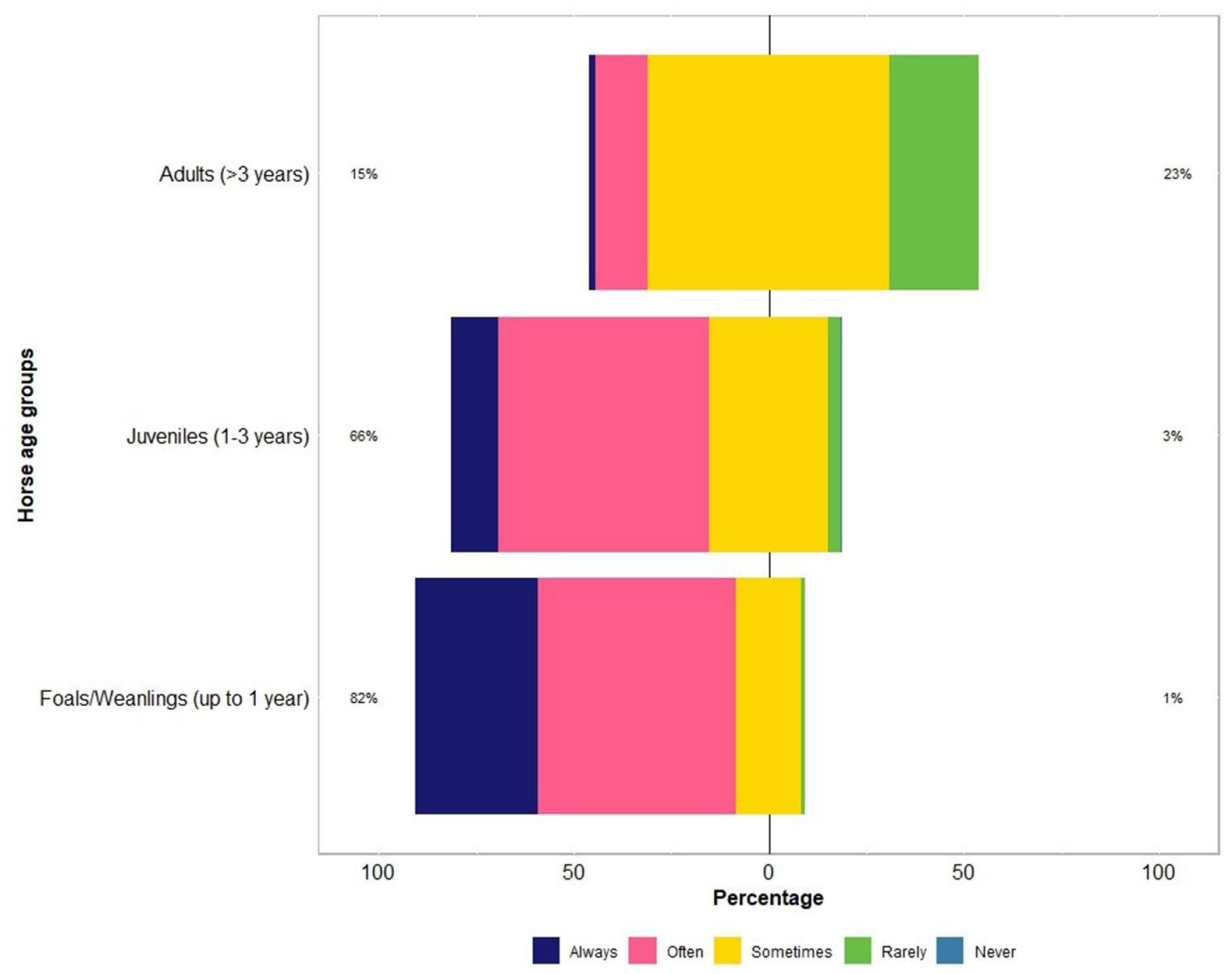
Percentage of respondents (*n* = 118) reporting their knowledge on the risk of worms in different age groups of Australian horses using a Likert scale.

**Figure 2:**
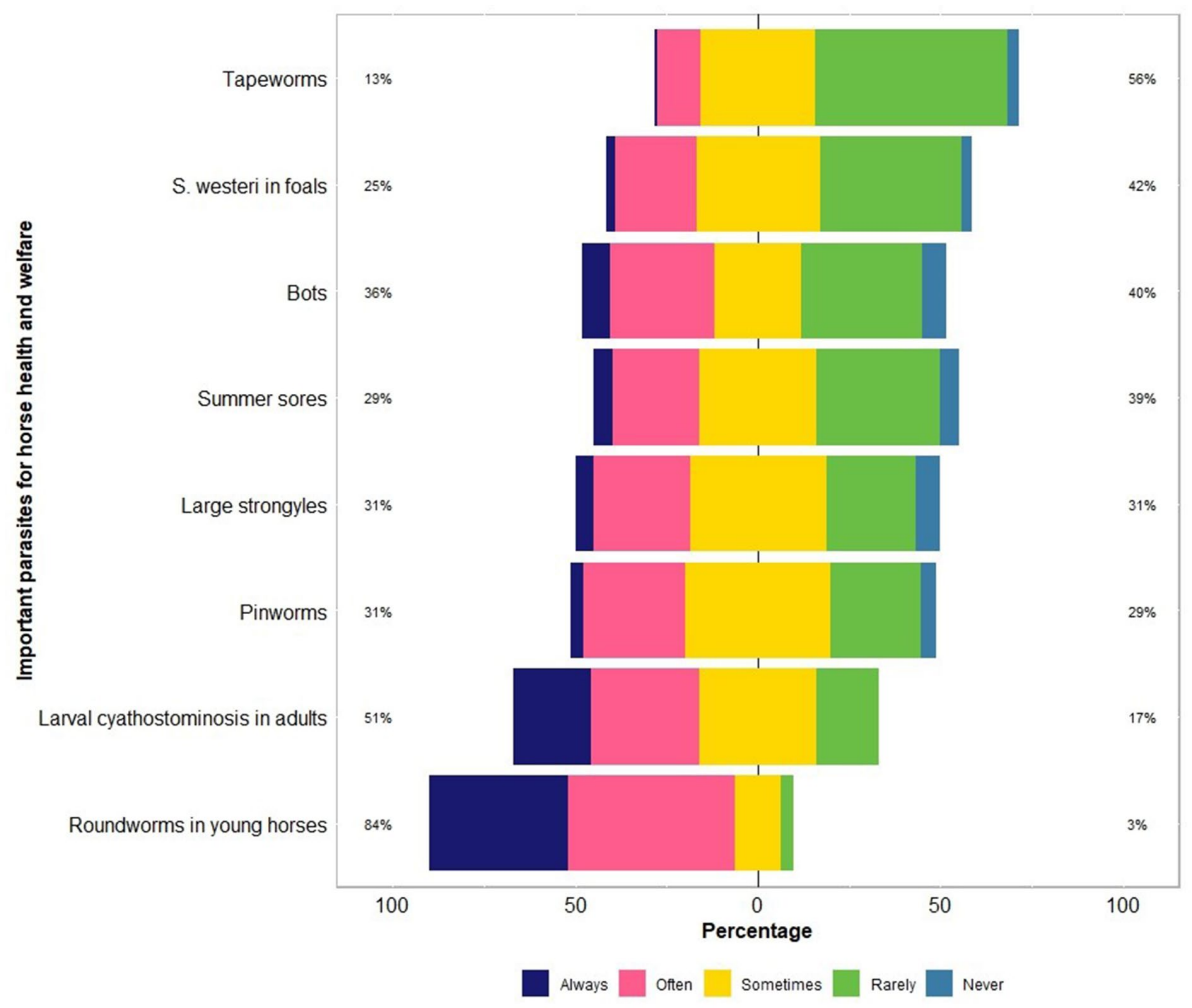
Percentage of respondents (*n* = 118) reporting their knowledge of parasites which are important for the health and welfare of Australian horses using a Likert scale.

**Figure 3:**
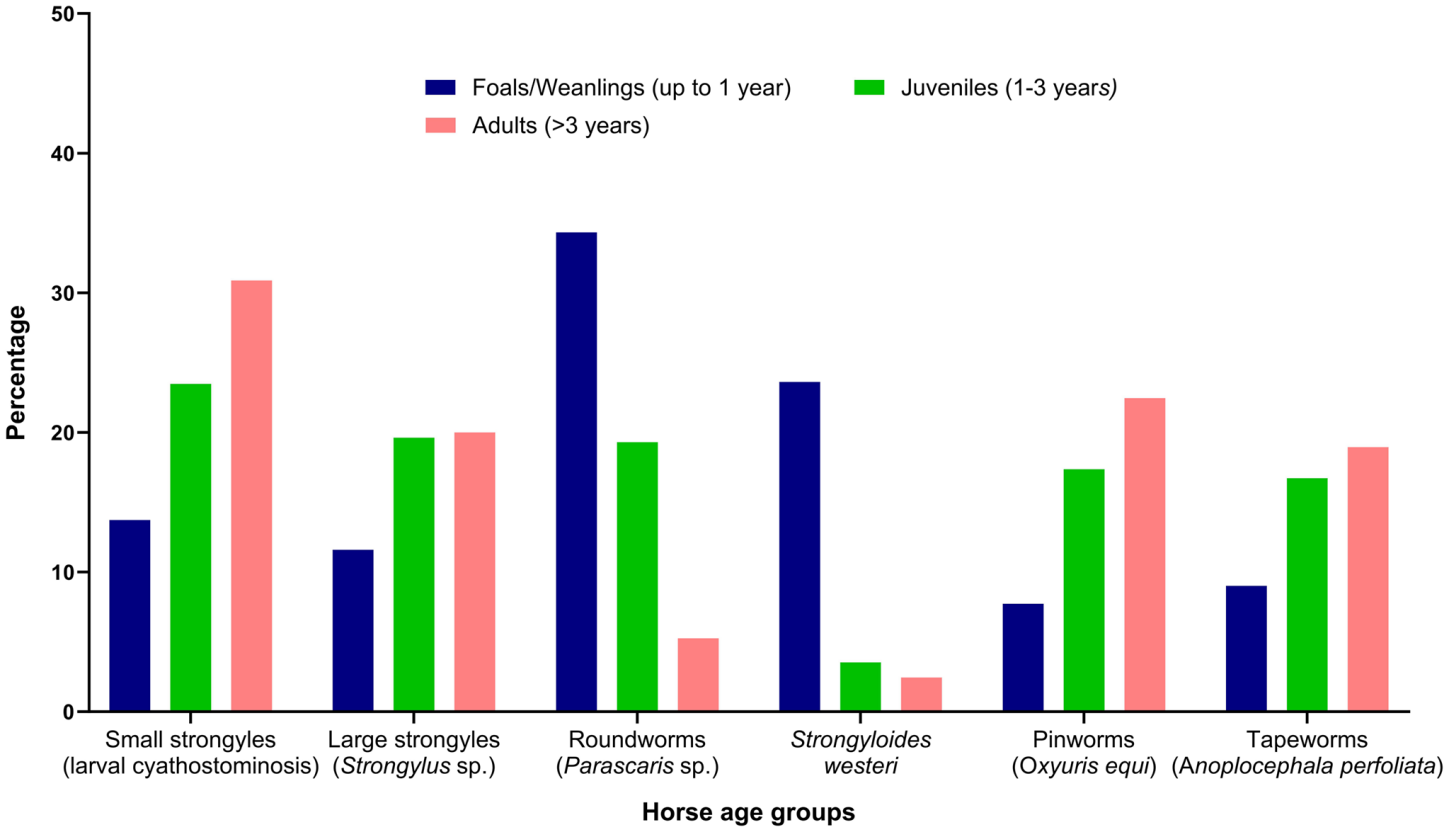
Percentage of respondents (*n* = 118) reporting their knowledge of the risk of various gastrointestinal nematodes to different age groups of Australian horses.

The respondents were confident (mean = 60; median = 64 on an analogue scale ranging from 0 to 100) about their knowledge of equine parasites (Table 2). However, within the last three years, only 39% (46 of 118) had attended a continuing professional development course on horse parasite control. Of 118 respondents, most of them reported that the occurrence of clinical parasitism varied in different age groups of horses, with foals (82%) and juveniles (66%) being always or often more susceptible to parasites than adults (15%) (Figure 1). In terms of the importance of worms in horse health and welfare, respondents ranked roundworms (i.e., *Parascaris* spp.) in young horses (i.e., <1 year; 84%, 99 of 118) and larval cyathostomins in adults (51%, 60 of 118) to be the most important (Figure 2). Furthermore, the respondents reported that *Parascaris* spp. (34%, 80 of 233) and *Strongyloides westeri* (24%, 55 of 233) were the two most important worms for foals and weanlings, whereas ≥20% of the respondents indicated the significance of cyathostomins and *Strongylus* spp. in juveniles and adult horses (Figure 3).

### 3.3. Diagnosis of worms

Most of the respondents (69%, 81 of 118) had examined horses with clinical parasitism in the last year, mostly in spring (29%, 41 of 142) followed by autumn (24%, 34 of 142), summer (11%, 16 of 142) and winter (8%, 12 of 142), with approximately one-quarter (24%, 34 of 142) observing no seasonal pattern. The main clinical signs associated with parasitism observed were weight loss or ill-thrift (16%, 66 of 418), followed by colic (14%, 60 of 418), tail rubbing (14%, 58 of 418), worms in the faeces (13%, 54 of 418) and diarrhoea (12%, 52 of 418). In addition, other signs, including rough coat (12%, 51 of 418), pot-bellied appearance (10%, 42 of 418), anaemia (4%, 18 of 418), and coughing or nasal discharge (3%, 12 of 418) were also ascribed to parasitism in horses by respondents. Although 97% (114 of 118) of the respondents reported recommending FECs for parasite management to their clients, only 40% (96 of 239) used FECs to diagnose GINs. Other diagnostic methods used were a response to treatment (21%, 49 of 239), clinical pathology results (15%, 35 of 239) and faecal culture (10%, 23 of 239) (Table 3). In addition, FECs were used for making deworming decisions (29%, 79 of 277), worm monitoring (26%, 73 of 277), efficacy of anthelmintics (26%, 73 of 277) and laboratory diagnosis of suspected cases of parasitism (18%, 51 of 277). Most of the respondents recommended FECs every 3 to 6 months (53%, 62 of 118). Faecal samples were mainly collected from all age groups of horses (49%, 67 of 118) and tested at a veterinary clinic (51%, 61 of 119) or diagnostic laboratory (44%, 52 of 119) (Table 3).

**Table 3:** Use of diagnostic methods and anthelmintics recommended by respondents for gastrointestinal nematode infections in horses.

Question (total responses)	Level	Percentage (counts)
Diagnosis of worms (*n* = 239)^a^	Faecal egg count	40 (96)
	Response to treatment	21 (49)
	Clinical pathology results	15 (35)
	Faecal/larval culture	10 (23)
	Postmortem examination	8 (18)
	Surgery	4 (9)
	Clinical signs only	2 (4)
	Other	2 (4)
	None	0.4 (1)
Recommendation of faecal egg counts (*n* = 118)	Yes	97 (114)
	No	3 (4)
Purpose of faecal egg counts (*n* = 277)^a^	Deworming decision	29 (79)
	Monitoring	26 (73)
	Effectiveness of anthelmintics	26 (73)
	Suspected parasitism	18 (51)
	Other	0.4 (1)
Frequency of faecal egg counts (*n* = 118)	Every month	1 (1)
	Every 2 months	4 (5)
	Every 3 months	23 (27)
	Every 4 months	9 (10)
	Every 6 months	30 (35)
	Once per year	14 (17)
	Egg re-appearance period data	13 (15)
	Other	7 (8)
Animals tested for faecal egg counts (*n* = 138)^a^	Foals/Weaning (up to 1 year)	11 (15)
	Juveniles (1–3 years)	17 (23)
	Adults (>3 years)	17 (24)
	Sick animals	7 (9)
	All horses on the farm	49 (67)
Testing of faecal samples (*n* = 119)^a^	Own veterinary clinic	51 (61)
	Diagnostic laboratory	44 (52)
	On farm	1 (1)
	Supplies store	2 (2)
	Other	3 (3)
Selection of anthelmintics (*n* = 118)	Anthelmintic active ingredient	48 (57)
	Faecal egg counts	20 (23)
	Combinations of anthelmintics	12 (14)
	Peer-reviewed literature	11 (13)
	Rotation of anthelmintics	3 (4)
	Accessibility	3 (3)
	Experience	2 (2)
	Other	2 (2)

a Selection of multiple options was possible for these questions.

### 3.4. Anthelmintics and anthelmintic resistance

**Figure 4:**
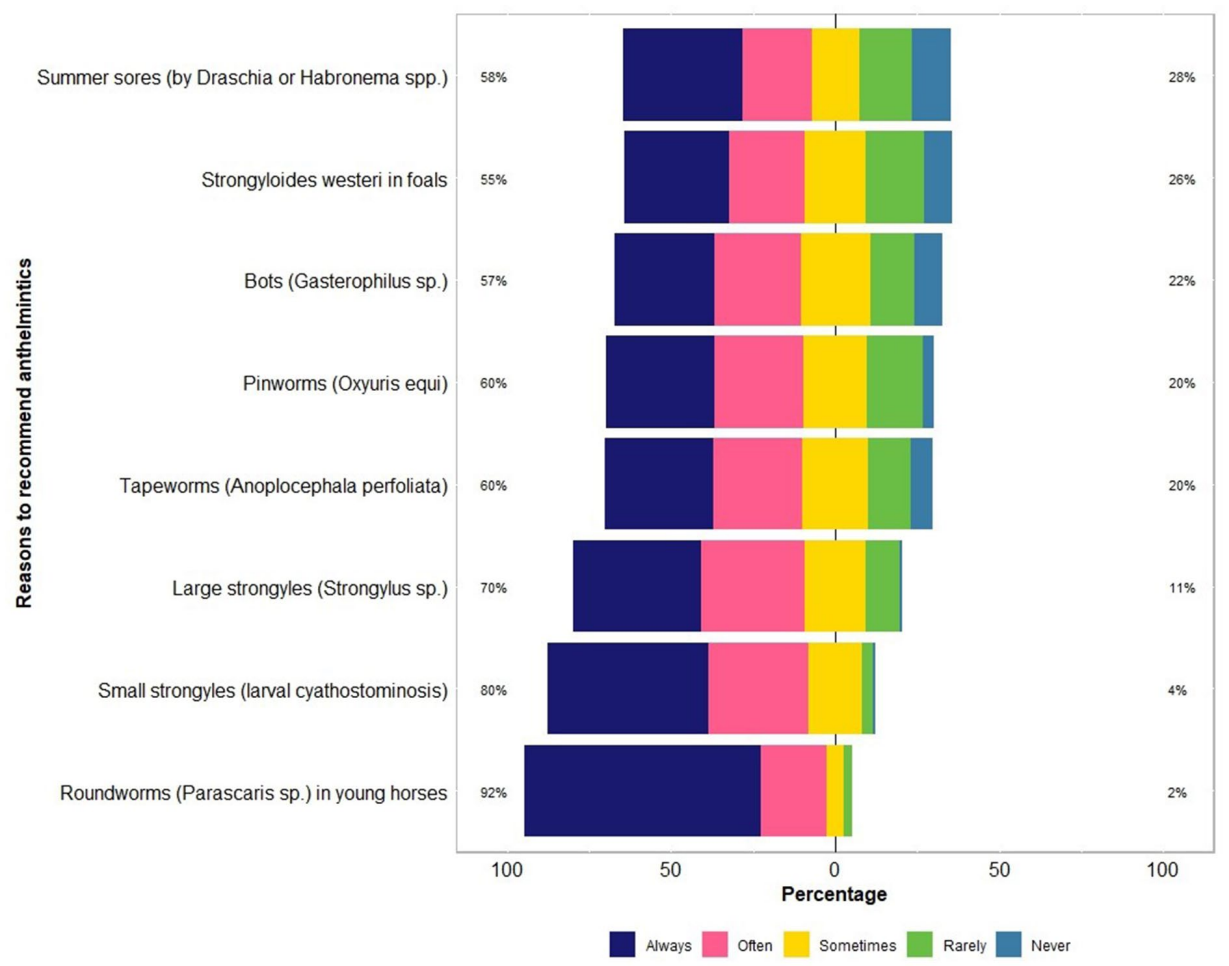
Percentage of respondents (*n* = 118) reporting their recommendations to use anthelmintics for controlling various types of parasites in Australian horses using a Likert scale.

The majority of respondents (97%, 115 of 118) recommended the use of registered anthelmintics for use in horses, always or often for the treatment and/or prophylaxis of *Parascaris* spp. (92%, 109 of 118), larval cyathostominosis (80%, 94 of 118), strongylins (large strongyles; 70%, 83 of 118), *Strongyloides westeri* (55%, 65 of 118), summer sores caused by *Draschia* and *Habronema* spp. (58%, 68 of 118), pinworms (60%, 71 of 118) and tapeworms (60%, 67 of 118) (Figure 4). In addition, 9% (11 of 118) of the respondents recommended BioWorma^®^ (fungal spore-based biological control product, https://www.bioworma.com/bioworma).

**Figure 5:**
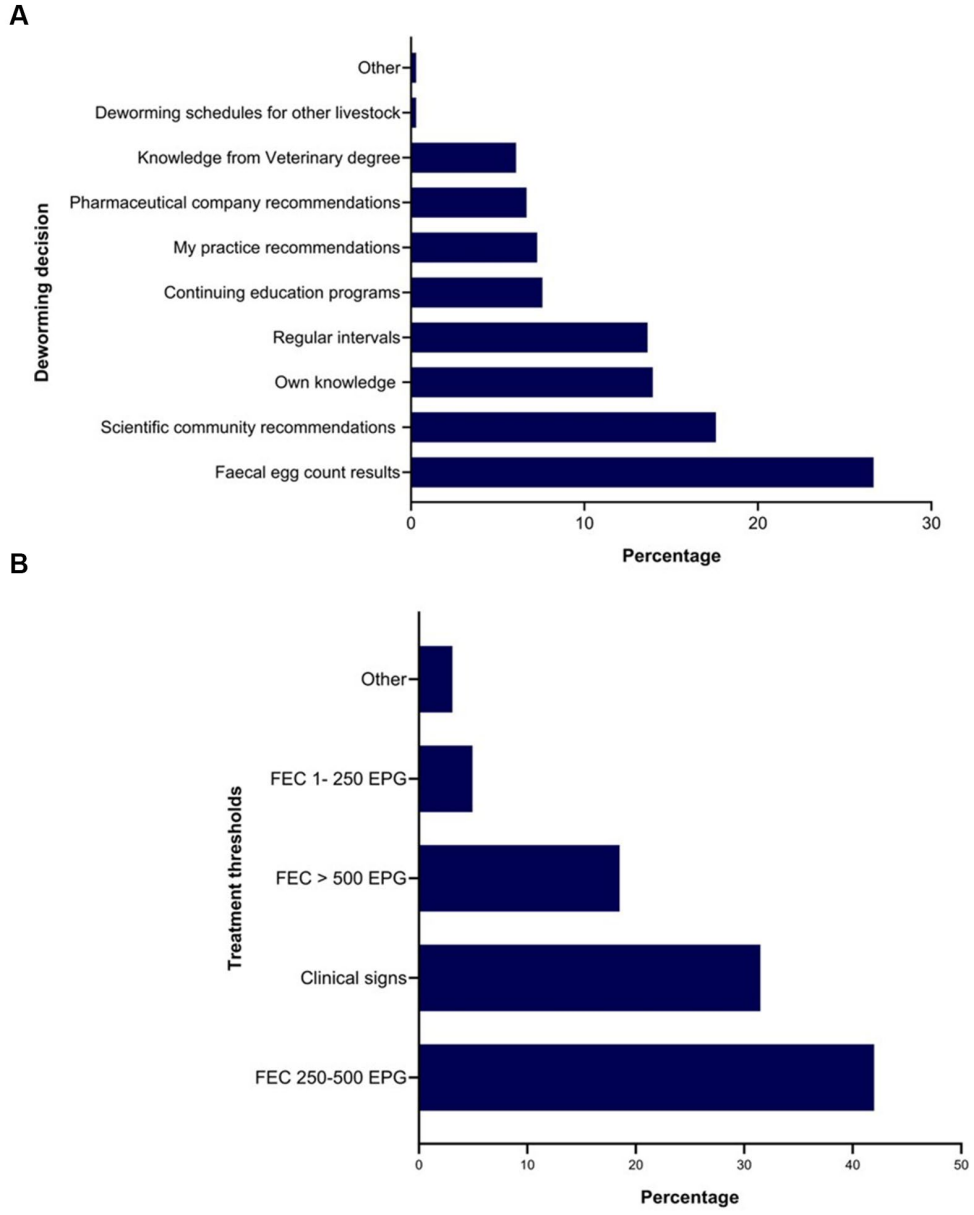
Percentage of respondents (*n* = 118) reporting the rationale for recommending anthelmintics **(A)** and the use of faecal egg thresholds for treating Australian horses **(B)**.

**Figure 6:**
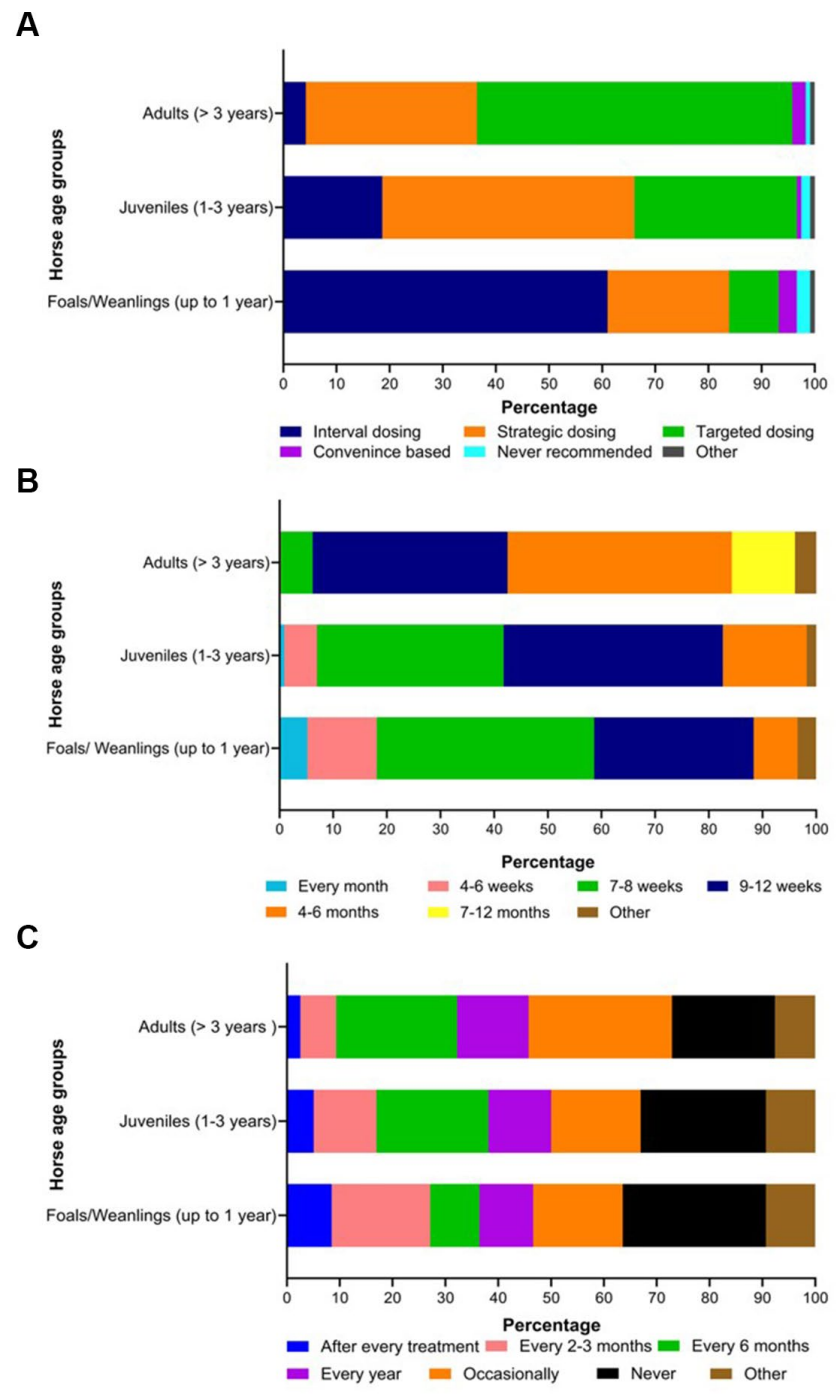
Percentage of respondents (*n* = 118) reporting their knowledge on the selection of deworming strategies **(A)**, the frequency of interval-based prophylactic anthelmintic treatments **(B)** and the rotation of anthelmintics **(C)** in various age groups of Australian horses.

**Figure 7:**
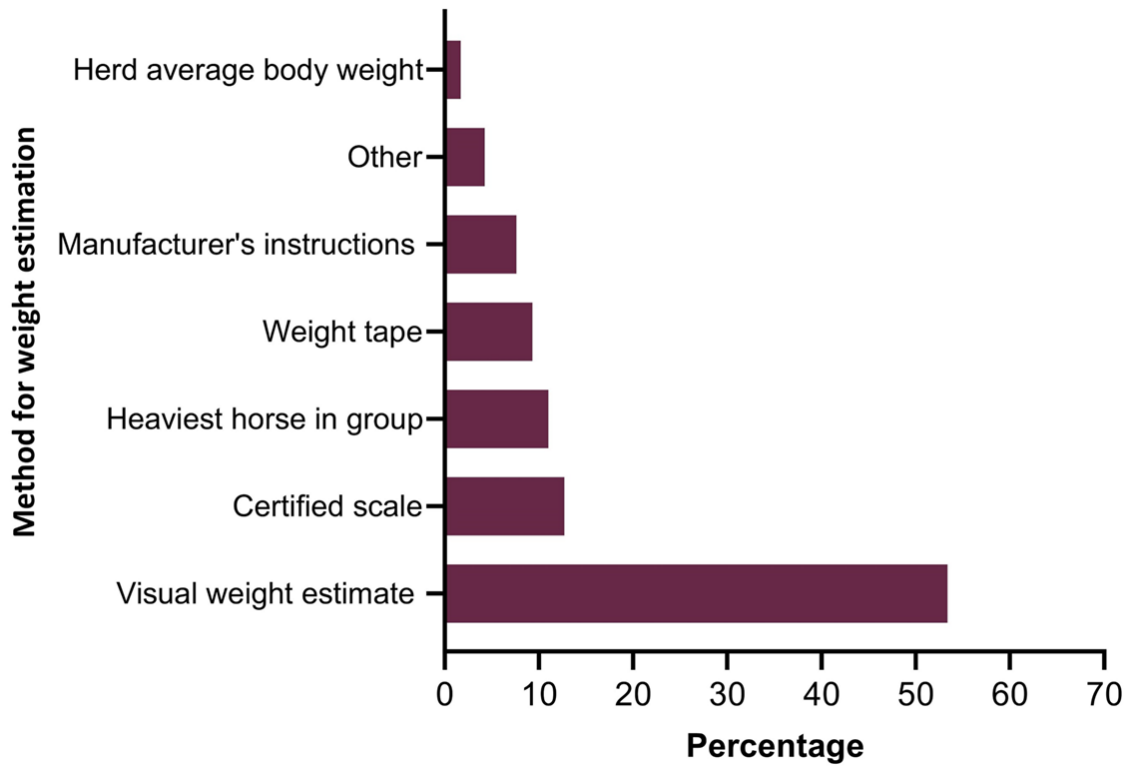
Percentage of respondents (*n* = 118) reporting the methods recommended for estimating the weight of horses to calculate the dose of anthelmintics.

Twenty-seven percent of the respondents (88 of 330) made their deworming decisions based on FECs followed by recommendations by scientific information (i.e., veterinary/veterinary parasitology books, peer-reviewed journals, guidelines for equine parasite control; 18%, 58 of 330), own knowledge (acquired during the veterinary degree) of respondents about GINs and regular intervals (14% each), continuing education programs (8%, 25 of 330) and respondents’ practice recommendations (7%, 24 of 330) (Figure 5A). To recommend deworming for horses, most respondents used a FEC threshold of 251–500 eggs per gram (EPG) of faeces (42%, 68 of 162) and clinical signs of parasitism (32%, 51 of 162) (Figure 5B). The top three reasons for the selection of specific anthelmintics were the type of anthelmintic drugs/class (i.e., active ingredient) (48%, 57 of 118), FECs (20%, 23 of 118) and products containing combinations of anthelmintics (12%, 14 of 118) (Table 3). The majority of respondents were using interval-based deworming for foals and weanlings (61%, 72 of 118) while strategic deworming (i.e., taking into account the parasite life cycle, climatic conditions, farm husbandry practices, etc.) for juveniles (47%, 56 of 118) and targeted deworming (i.e., based on faecal egg counts FECs) for adult horses (59%, 70 of 118) (Figure 6A), with 7 to 8 weeks interval being the most common for foals and weanlings (41%), 9 to 12 weeks for juveniles (41%) and 4–6 months for adult horses (42%) (Figure 6B). A few of the respondents were recommending frequent deworming of foals/weanlings (5%, every month; 13%, 4–6 weeks) and juveniles (6%, 4–6 weeks) while the rotation of anthelmintics was recommended by almost one-quarter of respondents (of 118) for all age groups of horses (Figure 6C). To calculate the dose of anthelmintics, visual estimate (53%, 63 of 118), weighing scale (13%, 15 of 118), actual/estimated weight of the heaviest horse in each age group (11%, 13 of 118), weigh tape (9%, 11 of 118) and manufacturer’s recommendations (8%, 9 of 118) were used to estimate the weight of horses (Figure 7). Most respondents (75%, 89 of 118) recommended deworming of visiting and/or new horses upon arrival at the property.

**Figure 8:**
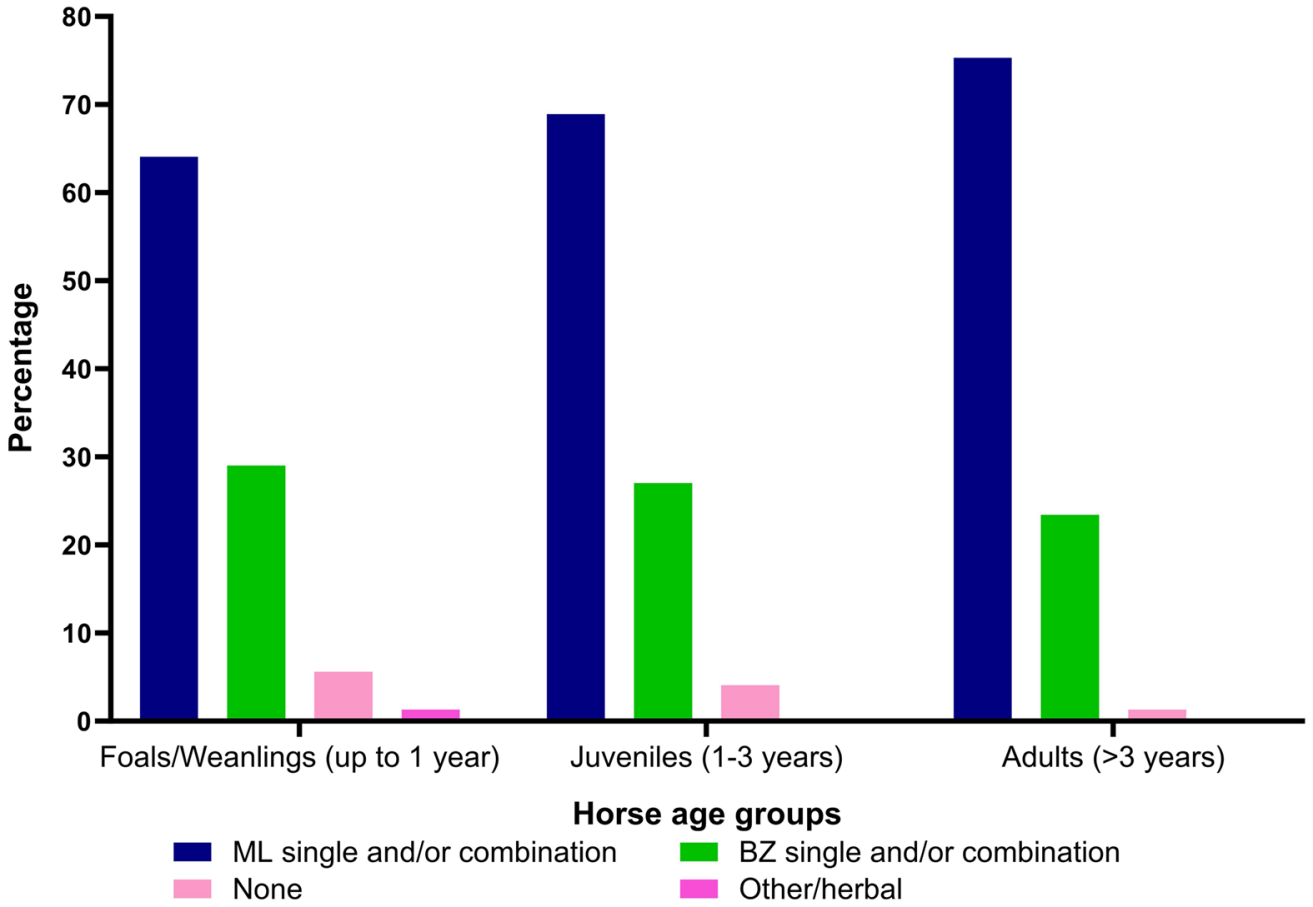
Percentage of the respondents (*n* = 118) reporting their recommendations on the use of anthelmintics (ML, macrocyclic lactones; BZ, benzimidazoles) in various age groups of Australian horses.

Macrocyclic lactones (e.g., ivermectin and moxidectin as single active or in combination with THPs, BZs, and/or praziquantel [PZL], 71%, 677 of responses) were the first choice of anthelmintics for all age groups of horses (foals/weanlings = 64%; juveniles = 69%; adults = 75%) while BZs (e.g., fenbendazole, oxfendazole as single or in combination with THPs and piperazine [PPZ] 27%, 184 of 677 of responses) were the second choice of anthelmintics (foals/weanlings = 29%; juveniles = 27%; adults = 23%) (Figure 8; Supplementary Table S1). Approximately 15% (18 of 118) of respondents reported deworming as an expensive component of the health management of horses while 81% (95 of 118) did not perceive it to be costly for their clients.

Most of the respondents (97%, 115 of 118) agreed that resistance to anthelmintics was a critical issue in managing GINs in horses, with 42% (50 of 118) being aware of AR on their clients’ properties, 40% (47 of 118) unaware and 18% (21 of 118) “did not know”. The majority of respondents (59%, 70 of 118) conducted and/or recommended the faecal egg count reduction test (FECRT) on their clients’ properties to assess the efficacy of anthelmintics and found resistance to MLs in most of the tests. As per veterinarians’ perceptions, AR had been observed in *Parascaris* spp. (30%, 38 of 118), cyathostomins (30%, 38 of 118), pinworms (16%, 20 of 118), strongylins (15%, 19 of 118), species of *Draschia* and *Habronema* (6%, 8 of 118), *Strongyloides westeri* (2%, 3 of 118) and tapeworms (1%, 1 of 118).

### 3.5. Husbandry practices and grazing management

More than 98% (116 of 118) of respondents believed that grazing management was an integral part of worm control in horses, and they recommended similar grazing management practices to their clients for both breeding (September to December) and non-breeding (January to August) seasons (see Supplementary Table S2). The most common grazing management practice recommended was rotational grazing and pasture hygiene (>30% of 118 respondents) for all age groups of horses. Furthermore, about 64% of the respondents (76 of 118) also recommended cross- or co-grazing of paddocks with other livestock species, including cattle (44%, 47 of 118), sheep (38%, 41 of 118), goats (13%, 14 of 118) and alpacas (5%, 5 of 118).

**Figure 9:**
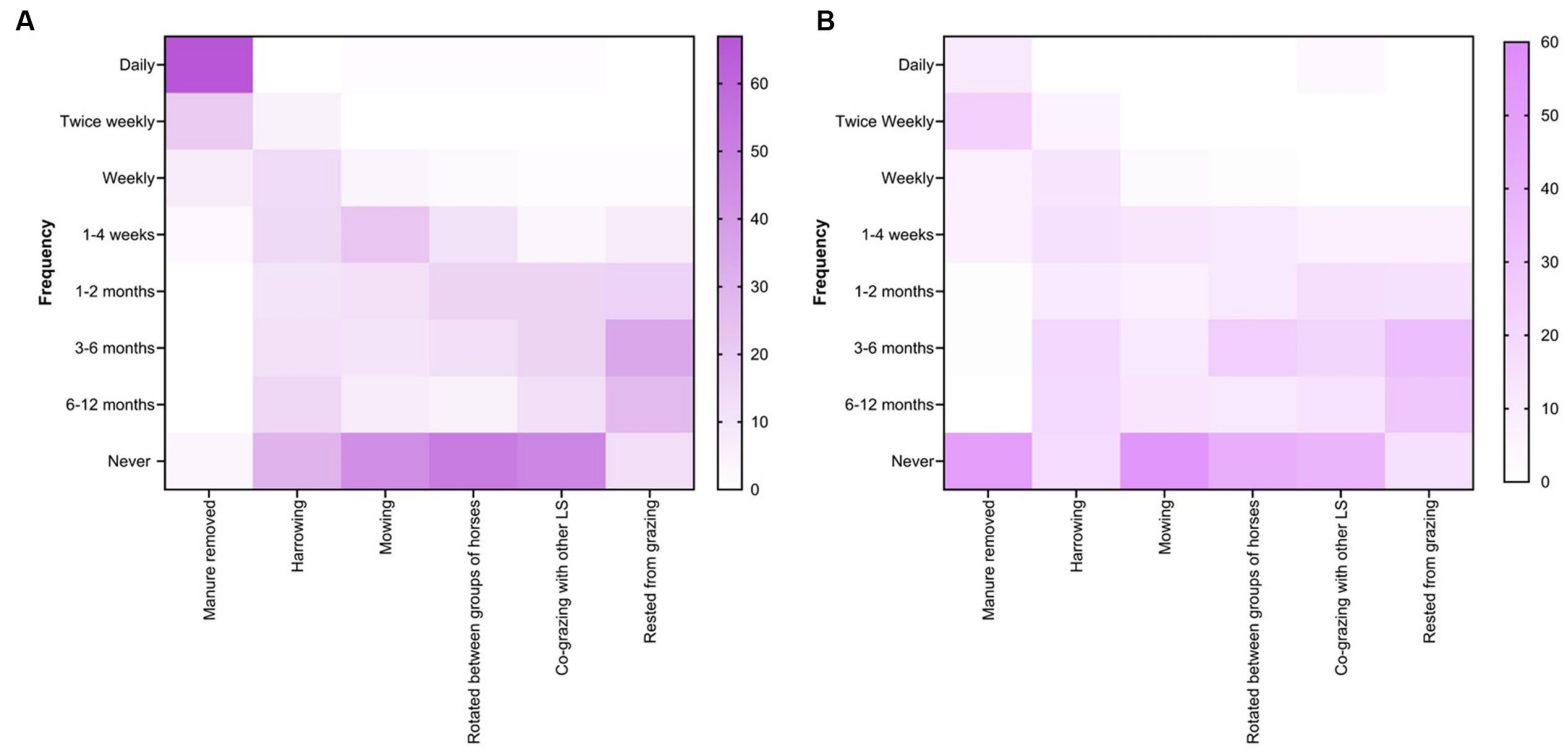
Percentage of respondents (*n* = 118) reporting their recommendations for the selection of various farm husbandry practices and grazing management for small **(A)** and large **(B)** paddocks at Australian horse farms.

Farm husbandry and pasture management practices were more regularly recommended by veterinarians on small paddocks (< ½ acre) (Figure 9A) than on large paddocks (> ½ acre) (Figure 9B). For instance, 67% (79 of 118) of respondents recommended daily manure removal from smaller paddocks whereas 49% (58 of 118) never recommended it for larger paddocks. Although the response rate varied (0 to 54%), most of the veterinarian’s recommended husbandry and management practices such as harrowing, mowing, rotations between horse age groups, co-grazing with other livestock species and pasture spelling from fortnightly to every 6 to 12 months to control worms in horses (Figures 9A,B).

### 3.6. Communication between veterinarians and clients on worm control

**Figure 10:**
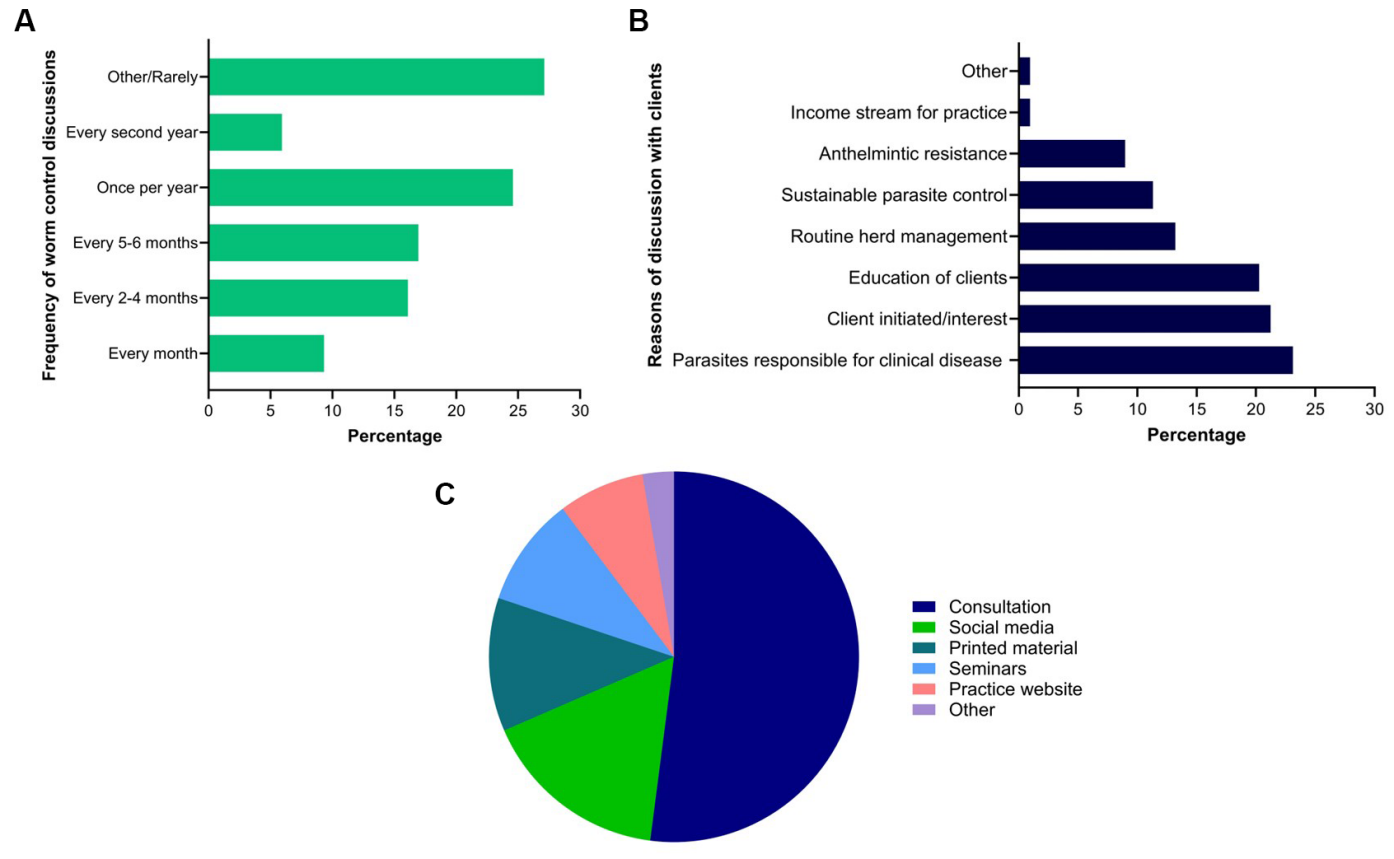
Percentage of respondents (*n* = 118) reporting the frequency **(A)** and reasons of their discussions with clients **(B)**, and methods used by them to educate their clients **(C)** regarding equine worm control practices in Australia.

Of the 118 respondents, approximately one quarter (27%, 32 of 118) rarely had discussions on worm control practices with their clients while others had it once per year (25%), every 2 to 4 or 5 to 6 months (each 17%), every month (9%), and biennial (6%) (Figure 10A). Respondents reported that such discussions were held mainly due to clinical parasitism (23%, 49 of 212), client-led (21%, 45 of 212) or for the education of clients (20%, 43 of 212) (Figure 10B). For the education of clients on worm control practices in horses, consultation was the most commonly used method (52%, 76 of 146) followed by social media (16%, 24 of 146), printed material (12%, 17 of 146), seminars (10%, 14 of 146) and the practice website (8%, 11 of 146) (Figure 10C). Similarly, the respondents relied on information which they acquired from guidelines published in scientific journals and professional organisations such as the Australian Association of Equine Practitioners (23%, 51 of 225), printed materials (18%, 40 of 225), seminars or conferences organised by the Australian Veterinary Association or Equine Veterinary Association (18%, 40 of 225), their veterinary course (14%, 31 of 225), continuing professional development courses (13%, 29 of 225), social media (4%, 8 of 225), webinars or podcasts (9%, 21 of 225) and discussion with other veterinarians or colleagues (2%, 5 of 225).

## 4. Discussion

This is the first study to understand Australian veterinarians’ knowledge, perceptions and strategies for worm control in horses. Overall, the findings illustrate that veterinarians have a good understanding of equine parasites and parasite control; however, there was a tendency to rely on frequent, interval-based prophylactic deworming in young horses and a sub-optimal adoption of FEC results as a basis for diagnosis of GIN infections (40%, 96 of 239) or deworming decisions (27%, 88 of 330) in all age groups of horses. Most of the respondents recommended MLs for all age groups of horses (71%, 481 of 677) and used visual estimation of the weight of animals to calculate the dose of anthelmintics (53%, 63 of 118). Although the majority of respondents (97%, 115 of 118) perceived AR to be a critical issue in managing worms in horses, 58% (67 of 118) of them were unaware of the status of AR on their clients’ properties. Forty-two percent (50 of 118) of the respondents perceived resistance in worms, including pinworms (16%), strongylins (15%), species of *Draschia* and *Habronema* (6%), *Strongyloides westeri* (2%) and tapeworms (1%). These are important findings, as no such reports based on field studies to evaluate drug efficacy had been published at the time of the survey. However, recent field studies on Thoroughbred farms have reported anthelmintic treatment failures against *Triodontophorus brevicauda*, a species of large strongyles in Australia (10) and a horse tapeworm, *Anoplocephala perfoliata*, in the USA (37). The anecdotes reported by the respondents in this study warrant further investigations into the efficacy of commonly used anthelmintics against species other than ascarids and cyathostomins, particularly on Thoroughbred farms where frequent, prophylactic interval-based deworming is a common practice.

Despite the novelty of this study, results should be carefully interpreted due to the following limitations. In this study, the response rate of the questionnaire was 10%, despite repeated reminders from EVA to its members over 1 year. This low response rate, potentially influenced by survey fatigue, could lead to bias due to over- or under-presentation of some of the responses. Additionally, selection bias could impact the results as those veterinarians actively promoting parasite control among their clients may have been more likely to participate. Obsequiousness bias, where those being questioned alter their responses in a way they perceive to be desired by the investigator, may also have impacted the internal validity of this study. Another form of bias could have arisen from missing data. Imputation methods have been widely used to impute missing data from surveys conducted in both health (38) and agriculture (39). Most of such studies have preferred the imputation of missing data over deletion or mean replacement (39).

In this study, most (>65%) respondents believed that younger horses were more susceptible to worms than adult horses. Respondents ranked *Parascaris* spp. and *Strongyloides westeri* and cyathostomins as the most important parasites of young horses and adult horses, respectively. These findings concur with veterinarians’ perceptions worldwide regarding the importance of various worms in different age groups of horses (29, 32, 40). Interestingly, *Strongylus* spp. and *Oxyuris equi* were ranked equally important (31%) by Australian respondents, while surveys conducted in Denmark and France (29, 32) found strongylins were considered important, and *O. equi* was not. The reason for the discrepancy between perceptions of Australian and overseas veterinarians on the occurrence of *O. equi* remains unclear as this parasite has a global distribution and is found on every continent where horses are present (41). Approximately 13% of respondents perceived tapeworms as important parasites in horse health and performance which is similar to the findings of other studies on veterinarians’ perceptions of equine parasite control (29, 32). This could be due to the low detection limit of the routine diagnostic method (i.e., modified McMaster technique) (42) or the routine administration of anthelmintic products containing praziquantel used at the majority of the Australian horse properties (16).

We found that 97% (114 of 118) of the respondents recommended the use of FECs for parasite diagnosis every 3 to 6 months (53%) and prescribed different deworming strategies based on age groups of horses, including interval-based for foals and weanlings (61%), strategic for juveniles (47%) and targeted for adult horses (59%). Furthermore, 42% (68 of 162) of the respondents recommended deworming using the FEC threshold of 251–500 EPG. Previously, similar findings were reported in the UK where the majority of veterinarians (97%, 38 of 39) recommended FECs for the diagnosis of worms in horses and they also considered the FEC results before making deworming decisions (43). However, a French study reported that almost half of 91 surveyed veterinarians never used FECs for deworming decisions and relied on “blanket” deworming (32). A surveillance-based targeted drenching strategy has been suggested as a preferred approach to manage GINs in horses to slow the development of AR (4, 44), thereby maintaining a susceptible population of worms on pastures (refugia) to achieve sustainable control of worms (45). Recently, a simulation study demonstrated that targeted drenching based on FEC surveillance can delay the development of AR. This outcome was also linked to horse age and climatic conditions (46), indicating different deworming strategies for various age groups of horses to reduce the number of annual anthelmintic treatments, particularly in adult horses (4, 46). However, such strategies will require a greater number of FECs coupled with larval cultures per year to monitor *Strongylus* spp., the prevalence of which has been markedly reduced since routine deworming was introduced (4, 29, 47) in young (< 3 years) horses. Due to the pathogenicity of these species, ongoing surveillance is recommended when using selective or targeted deworming strategies, especially as the prevalence of *Strongylus vulgaris* in recent epidemiological studies was 7.8% in Australia (48), 61% in Sweden (49) and 79% in Italy (50).

More than two-thirds (71% of 677) of the responses received indicated recommendations for MLs (either in single active formulation or combined with other drug classes) to treat worms in all age groups of horses. Furthermore, only 42% (50 of 118) of respondents were aware of the status of AR on their clients’ farms. Recent studies on the epidemiology of GINs, worm control practices and the status of AR have revealed that MLs have been the first choice of horse managers, veterinarians and trainers globally to control GINs in horses (16, 18, 26, 27, 48, 51, 52). This preference for MLs could be due to the efficacy of this class (i.e., moxidectin) against cyathostomins (53, 54) which are the predominant parasites of horses and, therefore, the main target of horse parasite control programs. However, recent reports on the emergence of resistance to ivermectin and moxidectin in Australia (10, 12) and the USA (55, 56) are concerning as an over-reliance on MLs with frequent rotation of drugs within the same class might be further exacerbating the problem. Such parasite practices will potentially result in non-viable treatment options against GINs in horses (54). Therefore, parasite control strategies must incorporate non-chemical control methods such as grazing management, farm husbandry and biological control (BioWorma^®^) practices to achieve a level of sustainability.

In this study, 64% (of 118) of the respondents recommended co−/cross-grazing with sheep, goats, and/or cattle, and manure removal was more frequent in smaller paddocks (< ½ acre) than in larger paddocks (>1/2 acres). Previously, it has been shown that horses grazing on pastures containing manure are likely to be exposed to more significant numbers of infective larvae (57) whereas manure removal reduces the number of free-living parasitic stages in the environment, thereby reducing the number of anthelmintic treatments per year (58, 59). Recently, a French study compared FECs of strongylid nematodes in horses on farms with and without mixed grazing with cattle and found that mixed grazing resulted in low FECs due to the dilution effect, potentially due to ingestion of equine-specific parasitic larvae by cattle from the grazing pastures (60).

We found that most respondents recommended targeted drenching based on FEC surveillance for horses. However, 27% (32 of 118) rarely discussed equine worm control practices with their clients. In addition, such discussions occurred once a year (25%), mainly due to clinical parasitism (23%, 49 of 212) or client-led conversation (21%, 45 of 212). Although most respondents (mean = 60; median = 64) felt confident about their knowledge of equine parasites, only 39% (46 of 118) had attended a continuing professional development course on parasite control in horses in the last 3 years. In the absence of Australian horse-specific parasite control guidelines, 23% (51 of 225) of the respondents relied on information acquired from guidelines published in scientific journals and professional organisations like AAEP (13). It is important to note that these recommendations are prepared for horses in the USA, and might not be effective for other regions such as Australia due to different climates, herd management, various egg-shedding patterns and the composition of nematode species (10, 61–63). The findings of this study have highlighted the need for engagement between Australian veterinarians and their clients on the topic of equine GIN management and ongoing professional training to update knowledge on recent trends in anthelmintic resistance. The active participation of veterinarians in parasite management of horses could be achieved by implementing legislation for prescription-only use of anthelmintics in Australia as it has been previously in Austria, Canada (Quebec province), Denmark, Germany, Finland, Netherlands, and Sweden (64, 65). In Denmark, this approach has resulted in better uptake of selective and targeted deworming strategies and increased veterinarians’ involvement in formulating tailored worm control practices, ultimately resulting in more evidence-based control of equine parasites (30).

In conclusion, this study has provided insights into Australian veterinarians’ knowledge, perceptions and treatment strategies for managing equine parasites. The findings showed that some perceptions and practices related to veterinarians’ understanding of GINs and parasite importance in different age groups of horses, the diagnosis and control of worms, anthelmintics and AR, grazing management and communication between veterinarians and their clients on worm control are presumably contributing to the current status of AR in GINs of Australian horses. Based on these results, there is a need for regular monitoring of egg-shedding patterns in different age groups of horses and assessing the efficacy of anthelmintics to promote evidence-based parasite control programs. In addition, the legislation of prescription-only use of anthelmintics based on FEC results should be considered to deal with the critical issue of AR in the GINs of horses. Furthermore, ongoing professional development covering emerging trends in equine parasitology should be prioritised for veterinarians. In collaboration with other important stakeholders, education of clients on the effective management of horse worms can be achieved through a variety of platforms.

## Data availability statement

The original contributions presented in the study are included in the article/Supplementary material, further inquiries can be directed to the corresponding author.

## Ethics statement

The studies involving humans were approved by the Human Ethics Committee (Ethics ID 13193) of the University of Melbourne. The studies were conducted in accordance with the local legislation and institutional requirements. The participants provided their written informed consent to participate in this study.

## Author contributions

GA: Conceptualization, Data curation, Formal analysis, Investigation, Methodology, Visualization, Writing – original draft, Writing – review & editing. MS: Formal analysis, Writing – review & editing. JB: Conceptualization, Supervision, Writing – review & editing. AB: Conceptualization, Supervision, Writing – review & editing. CJ: Conceptualization, Writing – review & editing. CE-H: Conceptualization, Supervision, Writing – review & editing. EW: Conceptualization, Methodology, Writing – review & editing. PC: Conceptualization, Writing – review & editing. LC: Conceptualization, Writing – review & editing. JH: Conceptualization, Methodology, Writing – review & editing. IB: Conceptualization, Supervision, Writing – review & editing. MN: Conceptualization, Methodology, Writing – review & editing. KH: Conceptualization, Investigation, Methodology, Writing – review & editing. AJ: Conceptualization, Funding acquisition, Methodology, Project administration, Resources, Supervision, Writing – review & editing.

## Footnotes

^1^ https://www.ava.com.au/about-us/ava-groups/equine/

^2^ www.project-redcap.org

^3^ www.graphpad.com
