# Supplementary material for: Assessment of worm control practices recommended by equine veterinarians in Australia
Source: Front Vet Sci. 2023 Nov 3;10:1305360. doi: 10.3389/fvets.2023.1305360 (PMC10654783; doi:10.3389/fvets.2023.1305360)
Supplement: Supplementary file 1 [file Table_1.DOCX]

Supplementary Table 1. Anthelmintics recommended by respondents in various age groups of Australian horses.

| Dewormer(s) used | Responses (%) | | |
| --- | --- | --- | --- |
|  | Foals/Weanlings | Juveniles | Adults |
| Macrocyclic lactone ^a^ | 7 | 6 | 11 |
| Macrocyclic lactone + Praziquantel | 43 | 59 | 74 |
| Macrocyclic lactone + Tetrahydropyrimidines ^b^ | 6 | 8 | 10 |
| Macrocyclic lactone + Tetrahydropyrimidines + Praziquantel | 7 | 8 | 5 |
| Macrocyclic lactone + Benzimidazole ^c^ + Praziquantel | 2 | 2 | 3 |
| Benzimidazole | 14 | 15 | 13 |
| Benzimidazole + Tetrahydropyrimidines | 14 | 16 | 18 |
| Benzimidazole + Piperazine | 1 | 2 | 1 |
| None | 6 | 5 | 2 |
| Other | 1 | 0 | 0 |

^a^ Abamectin, Ivermectin, Moxidectin

^b^ Pyrantel embonate, Morantel tartrate

^c^ Oxfendazole, Oxibendazole, Fenbendazole

|  | No. of foals/Weanlings (up to 1 year) | |  | No. of juveniles (1-3 years) | |  | No. of adults (> 3 years) | |  |
| --- | --- | --- | --- | --- | --- | --- | --- | --- | --- |
| Grazing management practices (%) | Breeding season | Non-breeding season | *P value* | Breeding season | Non-breeding season | *P value* | Breeding season | Non-breeding season | *P value* |
| Break feeding | 3 | 4 | 1.00 | 1 | 0 | 1.00 | 4 | 1 | 0.37 |
| Rotational grazing | 48 | 48 | 1.00 | 64 | 52 | 0.46 | 39 | 38 | 1.00 |
| Set stocking | 7 | 8 | 1.00 | 3 | 8 | 0.11 | 6 | 7 | 0.78 |
| Pasture hygiene | 39 | 36 | 0.77 | 31 | 28 | 1.00 | 47 | 50 | 0.67 |
| Other | 3 | 4 | 1.00 | 2 | 2 | 1.00 | 4 | 3 | 1.00 |

Supplementary Table 2. Grazing management practices recommended by respondents (*n* = 118) during breeding and non-breeding seasons for Australian horses.
